# Advancements in Targeted Therapies for the Management of Crohn’s Disease: A Comprehensive Review

**DOI:** 10.7759/cureus.61751

**Published:** 2024-06-05

**Authors:** Peter Girgis, Tanisha LNU, Amna Ahmad, Mina Daniel, Maria Kamel, Jade L Gambill, Atika Shahzadi, Usman Khan, Anam Zara, Vagisha Sharma

**Affiliations:** 1 Internal Medicine, Ross University School of Medicine, Bridgetown, BRB; 2 Department of Internal Medicine, Bogomolets National Medical University, Kyiv, UKR; 3 Gastroenterology, Rashid Latif Medical College, Lahore, PAK; 4 Internal Medicine, Memorial Hermann The Woodlands Medical Center, The Woodlands, USA; 5 General Physician, Independent Researcher, Houston, USA; 6 Neuroscience, Parker University, Dallas, USA; 7 Medicine, Aziz Bhatti Shaheed Teaching Hospital, Gujrat, PAK; 8 General Practice, Akhtar Saeed Medical and Dental College, Lahore, PAK; 9 Medicine, HCA Houston Healthcare Kingwood, Houston, USA; 10 College of Medicine, Vardhman Mahavir Medical College and Safdarjung Hospital, New Delhi, IND

**Keywords:** jak inhibitors, integrin inhibitors, biologics, crohns disease, ibd

## Abstract

Crohn’s disease (CD) is a complex clinical condition characterized by persistent gastrointestinal inflammation that leads to episodes of flare-ups and subsequent healing. The treatment options for this disease are heterogeneous as its impact on different patients is also different. This study aims to evaluate the effectiveness of recently approved drugs that specifically target certain pathways within cells that are involved in CD pathogenesis. These medicines include biologics like anti-TNF agents, interleukin inhibitors, and small molecule inhibitors; they work by altering the modulation of immune responses and reducing inflammation. These drugs seem promising in terms of inducing remission in moderate to severe CD among various patient populations. Conversely, it is possible to examine how well these drugs perform using gene expression and molecular markers. By understanding these results along with other ongoing trials, personalized medicine can be used more frequently by doctors who will adopt a strategy for an individual patient, maximizing benefits while minimizing adverse effects. There are still some issues that need to be worked out like the high cost associated with these drugs or immunogenicity risk and infectious complications too. In conclusion, there has been a remarkable improvement in CD management over the past decade with customized drugs leading toward a precision medical era. Further understanding of molecular mechanisms implicated in CD pathogenesis and new therapeutic approaches could potentially improve treatment outcomes among affected individuals. This research is crucial in understanding how CD therapeutics are changing, thus facilitating selection by doctors on the most appropriate methods for individualized patient care.

## Introduction and background

Crohn's disease (CD) is a multidimensional inflammatory bowel disease that results in a myriad of inflammatory processes in the gastrointestinal tract. These include the clustering of immune cells known as granuloma formation and the inflammation of all layers of the wall of the digestive tract, known as transmural inflammation [1]. The disease is often characterized by deeper bowel layers involvement, and several parts of the digestive tract may be affected. It usually has a relapsing-remitting course with symptom-free periods interspersed with periods of severe symptoms. Symptoms range from fever, abdominal colic, fatigue, anorexia, weight loss, malnutrition, clinical-radiological signs of mechanical intestinal obstruction, or bloody diarrhea to even possible complications that can lead to death [2]. The incidence and prevalence of IBD are rising amongst ethnic groups, and this diversity needs to be investigated further to provide a more advanced approach to the treatment of the disease [1,2].

While the exact etiology of CD remains elusive, it is widely accepted that a combination of genetic predisposition, environmental factors, and dysregulated immune responses contribute to its pathogenesis. The latest international genome-wide association studies highlighting the genetic complexity of the disease have brought the number of IBD susceptibility gene loci to 163 [3]. However, genetic variants only explain a fraction of the disease heritability, further prompting the exploration of additional molecular mechanisms and gene-environment interactions contributing to CD susceptibility and progression. Epigenetics as a field has gained significant recognition due to its focus on regulating communication between the genome and the environment without changing the DNA sequence. Epigenetic modifications, like DNA methylation, histone acetylation, and non-coding RNA regulation, are dynamic and responsive to environmental triggers including diet, pollution, infections, and developmental signals [4]. This narrative review article aims to elucidate the complex interplay between epigenetic mechanisms and the development and progression of CD.

By summarizing the latest research and emphasizing key discoveries, the authors of this review hope to uncover epigenetics patterns that underlie this intricacy and the drugs that target these pathways to achieve disease remission. A thorough understanding of the pathogenesis of CD along with the identification of potential therapeutic targets based on epigenetic insights by this review could lead toward more precise medicine and better clinical outcomes for affected individuals.

Pathophysiology of CD

**Figure 1:**
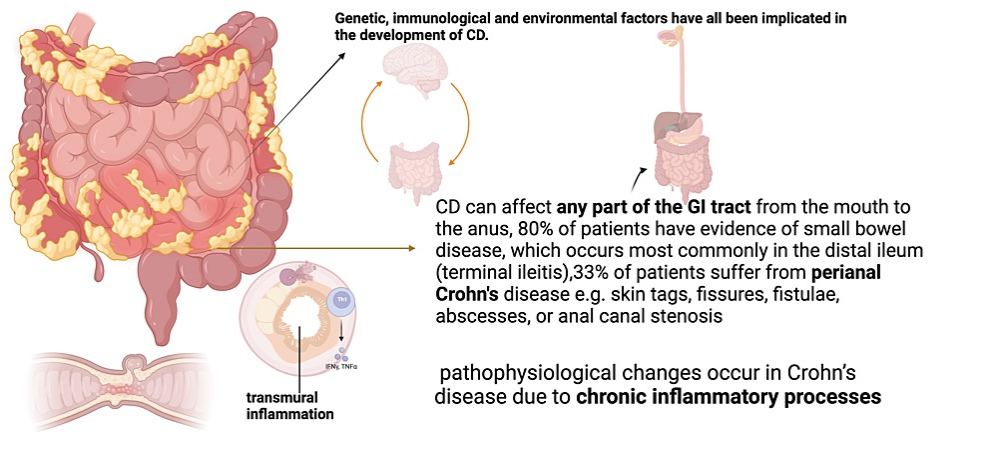
Pathophysiologic findings of Crohn's disease. References:  [5,6,8,9]. Image credit: Dr. Usman Khan. Created with biorender.com.

The pathophysiology of CD involves many factors, such as genetic predisposition, dietary, environmental, infectious, and immunological. The ensuing inflammation involves all layers of the gastrointestinal tract, thus called "transmural," and commonly involves the terminal ileum and right colon, but the entire gastrointestinal tract can be affected by this (Figure 1) [5]. The inflammation begins in the crypt, which is an invagination made by goblet, stem, and Paneth cells, leading to crypt inflammation and abscess formation. This eventually progresses to very small and shallow focal ulcers. These lesions then develop into deep longitudinal and transverse ulcers with intervening edema of the mucosa, giving the characteristic cobblestone pattern of the mucosa. Lymphedema, bowel wall, and mesenteric thickening usually occur due to the spread of inflammation in a transmural pattern. Bowel obstruction is another feature of CD that develops due to uncontrolled inflammation that results in hypertrophy of the muscularis mucosa, fibrosis, and eventually stricture formation (Figure 1) [6]. These areas are later replaced by scarring due to inflammatory flares [7]. Usually, adjacent structures like surrounding loops of the bowel, psoas muscles, and bladder get penetrated by fistulas that are formed during the progression of the disease. Sometimes, a fistula can even extend to the skin of the abdomen (anteriorly) or flanks as well [6]. One feature of CD is the formation of focal collections of macrophages, epithelioid cells, and multinucleated giant cells called granuloma. The granulomas in CD are noncaseating in nature, that is, they lack caseous necrosis. They can occur in any layer of the bowel wall, lymph nodes, liver, and peritoneum [6].

**Figure 2:**
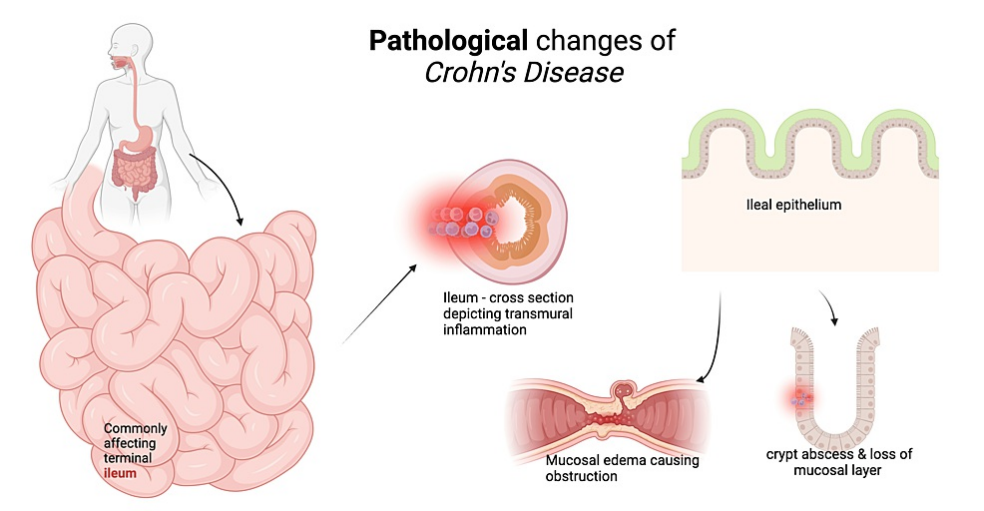
Pathological changes of Crohn's disease Image credit: Dr. Usman Khan [9]. Created with biorender.com.

CD is a form of IBD that has been linked to a genetic predisposition in correlation with the environment, as depicted in Figure 2. One genetic mutation that could be identified is a mutation in the nucleotide oligomerization domain containing the protein 2 gene (NOD2). This gene encodes a protein whose primary role is to protect the system against bacterial contamination; when this gene is mutated, it prevents Paneth cells from being able to appropriately discard bacteria, which then causes bowel lesions (Figure 1) [8,9]. Another gene that impacts CD is the autophagy-related 16-like 1 (ATG16L1), whose primary role is to regulate the autophagy pathway. A mutation in this gene would cause an increase in bacteria in the digestive tract due to the inability of Paneth cells to produce antimicrobial peptides [8]. Caspase recruitment domain 9 (CARD9) is another gene specializing in bacterial regulation in the gut microbiome. A mutation or a lack of this gene is linked with intestinal fungal dysbiosis [8]. These genes and others could significantly impact a patient's immune system and could serve as significant contributors to CD [9].

In addition, CD can be hallmarked by immunodeficiency [10]. The genetic variations mentioned impact the immune system by preventing the regular macrophage reaction to bacterial, viral, or fungal introduction (Figure 2) [9]. This lack of guard allows bacteria, fungi, and viruses to infiltrate the gut microbiome and either further prevent immune responses or cause direct damage to the intestinal system by eating away at the intestinal mucosa. Several microbiological organisms have, over the years, been linked to CD, such as* Mycobacterium paratuberculosis* (MAP), *Escherichia coli* (*E. coli*), and several others; however, there is little evidence and studies that can reliably state that these microbiological organisms are causative of CD and not just correlative (Figure 2) [9]. An additional immune response that is depleted in patients with CD is the permeability of their intestinal mucosa. Some studies have shown that patients with CD have an intestinal mucosa that is easy to permeate, which can cause complications, such as intestinal ulcers [9]. The immune disruptions could strongly impact a patient's ability to digest all foods appropriately and in turn could lead to CD [9] (Figure 2).

## Review

Methodology

A comprehensive review of articles discussing pharmacokinetics, pharmacodynamics, and clinical trial results of newly approved drugs for CD management and current therapies from 2014 to 2024 was performed. Search engines, such as PubMed, Google Scholar, and Medline, were used. The following terms were used in our searches using the boolean algorithm: (Crohn's Disease) OR (Inflammatory Bowel Disease) AND (management) OR (clinical trial) OR (targeted therapy) OR (biologics). We considered those articles that are in English and were published between 2014 and 2024. These included laboratory research, clinical trials, systematic reviews, and randomized controlled trials with free full text available. We excluded books studies, non-randomized controlled trials, and documents containing populations of children below 19 years old.

Current therapies used in the management of CD

**Figure 3:**
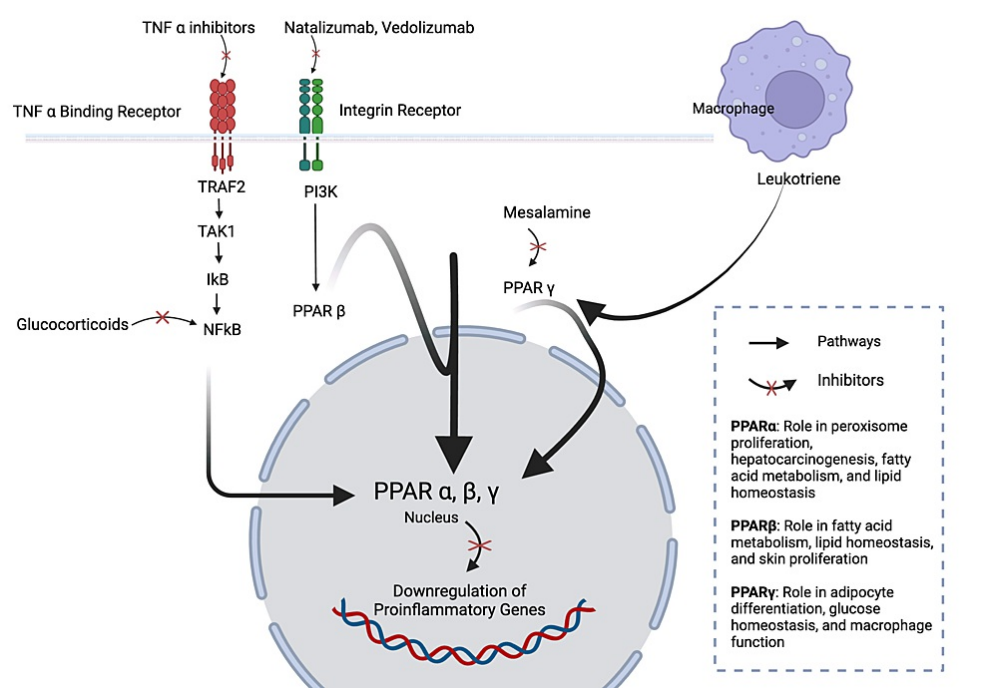
Overview of the mechanism of action of current therapies used in Crohn's disease management. References:  [12,13]. Image credit: Dr. Tanisha. Created with biorender.com.

Inflammation is the main culprit in the development of CD. Management focuses on sending these inflammatory processes into remission by utilizing monotherapy or combining several drugs, depending on the bandwidth of symptoms and flares. The aim of pharmacotherapy is the suspension of active symptoms, minimizing surgical load, and improving the overall well-being of the patient with CD [11]. An overview of the mechanism of action of certain drugs currently used in managing CD is shown in Figure 3.

Aminosalicylates

Aminosalicylates are a class of drugs that contain sulfasalazine and 5-aminosalicylic acid (5-ASA). Sulfasalazine stands out from other drugs of this class as it has a sulpha portion. The anti-inflammatory effect of sulfasalazine requires the enactment of gut bacteria [11,12]. Gut bacteria act on sulfasalazine and break it down into its metabolites: sulfapyridine and 5-ASA (mesalamine). Interestingly and particularly noteworthy is that the major anti-inflammatory properties of sulfasalazine are derived from 5-ASA [11,12]. It has also been proven to exhibit a lower degree of unwanted effects when compared to sulfapyridine. Therefore, to exploit this, in many clinical settings, the administration of 5-ASA alone is preferred as compared to sulfasalazine. However, 5-ASA is famous for its low absorption in the gut and systemic circulation [11,14]. It remains in the terminal ileum before finally being excreted out in stool. This property of 5-ASA can be exploited for imposing an anti-inflammatory effect topically on the colonic epithelium [11,14]. It is pivotal to understand that although to a lesser degree, 5-ASA does get absorbed in the gut. The absorbed 5-ASA is aggressively metabolized to metabolized to N-acetyl-5-ASA by N-acetyltransferase 1 (NAT1). NAT1 further binds to PPAR-gamma (peroxisome proliferator-activated receptor gamma), a nuclear hormone receptor, brings its conformational change, and leads its migration from the cytoplasm to the nucleus where it starts the domino effect of turning down the inflammatory casket [14]. In the nucleus, the conformational changes brought in PPAR-gamma due to the binding of NAT1 results in the employment of the co-activator, vitamin D3 receptor-interacting protein (DRIP) [11,14]. DRIP interacts with PPAR-gamma to form protein dimers. The consumption of PPAR-gamma in this process downregulates the PPAR-gamma response element (PPRE) down the line that ultimately sends the signal to switch off the inflammatory casket primarily by downregulating the nuclear factor kappa B (NF-kappaB), activated protein kinase (MAPK), ceasing COX-2 activity. This manifests as reduced cytokines and prostaglandin availability, which are vitals for the inflammation [11,14].

Novel agents with the above mechanisms to 5-ASA are currently underdeveloped. The ultimate goal for these agents is to minimize the unwanted effects in treatment by increasing precision and specificity toward PPAR-gamma. For example, the compound GED-0507-34 exhibits a 100- to 150-fold greater anti-inflammatory effect than 5-ASA [11,14]. A new generation of 5-ASA, balsalazide, can bypass the small intestine and release a high concentration of 5-ASA in the colon. The major limitation as shown by the repeated studies of sulfasalazine is that it only holds a slight upper hand when compared to placebo in the treatment of active CD of mild to moderate degrees and in the management of flairs [15]. Moreover, being a sulpha drug, it is not useful when treating CD in populations with sulpha drug allergies. All patients on sulfasalazine must be advised to promptly add folic acid supplementation to their diet. This holds special importance when treating women of childbearing age, as failure to do this can have disastrous effects on the fetus manifesting as neural tube defects [15]. Other than these concerns, the major adverse effects include hepatotoxicity, interstitial nephritis, and anemia (Table 1) [16].

**Table 1:** Aminosalicylates used in the management of CD, their efficacy, and adverse effects.

Aminosalicylates	Efficacy	Adverse effect
Olsalazine	Mild to moderate	Hepatotoxicity, headache, nausea, vomiting, anorexia [16]
Mesalamine	Active disease and maintenance, mild to moderate CD	GIT related, nausea, diarrhea, interstitial nephritis [16]
Sulfasalazine	Active disease and maintenance	SJS, hepatotoxicity, anemia [16]

Corticosteroids

Corticosteroids are drug classes that are molecular duplications of endogenous hormones produced by the adrenal gland. The hormones produced by the adrenal gland endogenously are broadly divided into glucocorticoids (the major glucocorticoid produced by the body is cortisol), mineralocorticoids (the major mineralocorticoid produced in the body is aldosterone), and androgenic sex hormones [17]. The synthetic forms of glucocorticoids are artificially administered to generate a plethora of desired outcomes in a CD setting, such as anti-inflammatory, anti-proliferative, suppression of overactive immune system, and constriction of vessels. It is noteworthy that the therapeutic effects of glucocorticoids are dose-dependent. It generally requires a higher dose to see the desired immunosuppressive effects [17]. The mechanisms by which this drug class exerts its anti-inflammatory and immunosuppressive effects are classified as genomic and non-genomic mechanisms. Genomic mechanisms are time-consuming. Glucocorticoid molecules are small in size. It is also highly lipophilic. Thus, these molecules can easily cross cell membranes and bind to intra-cytoplasmic glucocorticoid receptors [13,17]. Precisely, glucocorticoids bind to alpha isoforms of glucocorticoid receptors. This interaction results in the activation of these receptors, and the glucocorticoid-receptor complex translocates to the nucleus. In the nucleus, this complex acts on several DNA sites, rendering many of them inactive (transrepression) and switching down the bandwidth of gene transcription while activating (transactivation) many sites and upregulating anti-inflammatory genes. Nuclear factor-κB (NF-κB), activator protein-1, and interferon regulatory factor-3 gene transrepression lead to the suppression of pro-inflammatory cytokines and prostaglandin [13,17]. Transactivation of transcription factors, including glucocorticoid response elements (GREs), upregulates several anti-inflammatory cytokines. These include lipocortin1, NF-kB inhibitor, and IL-10. Meanwhile, the non-genomic mechanism is manifested immediately at higher doses of glucocorticoids due to its interaction with glucocorticoid receptors present on targeted cells (T lymphocytes). Glucocorticoids inactivate sodium and calcium channels present on these cells' membranes. This in turn brings down inflammation [13,17].

The main mechanism of corticosteroids in the treatment and prevention of CD remission is to put a halt to the transcription of interleukin and arachidonic acid metabolism, thus preventing the indispensable steps in the casket of inflammation and triggering lymphocyte apoptosis in the gut [13]. It is commonly employed after the failure of 5-ASA compounds. The commonly used corticosteroids include cortisone, prednisone, prednisolone, hydrocortisone, methylprednisolone, beclomethasone, and budesonide. However, the patient may experience adverse effects, such as Cushing syndrome, dyspepsia, diabetes, and/or osteoporosis [16,18]. Corticosteroids administered orally have proven efficacy in induction of remission. It has been the backbone of the treatment of CD since the 1950s. However, it is not equally efficient in the maintenance of remission in CD and is discouraged from being used for this purpose. The adverse effect profile of corticosteroids is extensive, particularly in the setting of comorbidity that can be worsened by glucocorticoid administration (Table 2). Other notable findings include includes hyperglycemia in the setting of diabetes [13,18].

**Table 2:** Adverse effects of corticosteriods

System	Adverse effects
Endocrine and metabolic system	Difficult glycemic control (particularly in patients with predisposed conditions, such as diabetes mellitus (DM)), Cushing's syndrome, and rapid withdrawal of glucocorticoids in patients on prolonged glucocorticoid therapy lead to adrenal insufficiency(hypotension, shock, hypoglycemia, and seizure) and growth delay in children [17,18].
Infections	Common bacterial, viral, and fungal pathogens due to the suppression of cytokines [16].
Cardiovascular system	Hypertension: higher dose, premature atherosclerosis [18].
Dermatological	Acne, hirsutism, facial erythema, impaired wound healing, skin thinning, and atrophy thinning of hair, perioral dermatitis, ecchymosis, and stria [18].
Ophthalmologic	Central serous chorioretinopathy, increased intraocular pressure leading to open angle glaucoma, bilateral cataract [13].
GI system	Gastritis, gastric ulcer, GI bleeding. Pancreatitis, visceral perforation, and hepatic steatosis (fatty liver). Rare: fat embolism and cirrhosis [13].
Neuropsychiatric	Mild euphoria or anxiety, hypomanic symptoms, depression with longstanding therapy, psychosis (exclusively with prednisone above 20 mg/day), akathisia, sleep disturbances [13].

The following assessment must be carried out before and during glucocorticoid treatment to analyze the risks: benefit ratio, dose and duration of treatment course, baseline monitoring (poorly controlled hypertension, cardiac failure with/without peripheral edema, low bone density/osteoporosis, psychiatric illness, systemic infection, peptic ulcer disease, and diabetes mellitus [18]), subsequent monitoring (assessment of bone health, assessment of hypothalamic-pituitary-adrenal (HPA) function, assessment of growth (children and adolescents), assessment of dyslipidemia and cardiovascular risk (adults), and assessment of ophthalmological complications [13,18].

Immunosuppressive Drugs

The goal of immunosuppressive therapy for the treatment of CD is to decrease the gut inflammatory process. Due to the pathophysiology of CD, a dysregulated, overactive immune response with an excess of pro-inflammatory cytokines has led to the relative importance of immunosuppressive therapy. These drugs work by reducing the function of immune cells. Furthermore, suppressing gut inflammation plays a pivotal role in reducing flares, but therapy may take up to several months before they take effect [19]. Among the immunosuppressants, specific drug therapy is highly selective and accounts for disease severity, frequency of flares, and the goal to maintain remission. 

Azathioprine (AZA)/6-Mercaptopurine(6-MP)

AZA is a prodrug of 6-MP. Both of these drugs aim at converting 6-thioguanine (6-TG), into inactive metabolite, 6-thiouric acid, by xanthine oxidase, and to the hepatotoxic metabolite 6-methylmercaptopurine (6-MMP) by the thiopurine methyltransferase (TPMT) enzyme [15,19]. Its primary mechanism is to add the active drug metabolites into cellular nucleotides, which imparts anti-inflammatory effects by ceasing T cell proliferation and natural killer cell activities. This treatment option is used in CD to provide relief by controlling gastrointestinal symptoms, such as diarrhea, gastrointestinal bleeding, and pain [15]. This is particularly useful in perianal and fistulising CD management. It is primarily used as a maintenance therapy but is not used for induction of remission. It can take up to 12 weeks for results to become effective. Furthermore, xanthine oxide inhibitors can aid in a stronger response, in some patients who tend to shunt toward 6-MMP. The adverse effects can be severe and include pancreatitis, hepatotoxicity, and bone marrow suppression(Table 3) [19].

**Table 3:** Adverse effects of immunosuppressive drugs

Immunosuppressive drugs	Adverse effects
6-mercaptopurine (6-MP) and azathioprine (AZA)	Leukopenia, hypersensitivity reaction (hepatitis, pneumonitis, arthritis, and fever), pancreatitis, minor adverse effects (nausea, vomiting, and flu-like illness), opportunistic infections. Rare: increase in the risk of non-melanomatous skin cancer hepatosplenic T cell lymphoma (HSTCL) [15,16].
Methotrexate (MTX)	Leukopenia, hepatotoxicity, hypersensitivity pneumonitis, and opportunistic infections [15,16].
Cyclosporine (CSA)	Anaphylaxis, seizure, pneumocystis carinii pneumonia, and permanent nephrotoxicity [16].

Methotrexate (MTX)

The mechanism of action of methotrexate is to inhibit dihydrofolate reductase. Dihydrofolate reductase plays a pivotal role in the metabolism of folic acid, thus ultimately inhibiting the production of deoxyribonucleic acid (DNA), ribonucleic acid (RNA), and protein [15,19]. Its effect also comes from its ability to inhibit folate enzyme-dependent immunomodulation. MTX is used in patients with CD who remain unresponsive to other immunosuppressants. It should be used cautiously due to its adverse effects, such as hepatotoxicity, leukopenia, and pneumonitis (Table 3) [15,19].

Cyclosporine

This cyclosporine is an immunomodulator that targets T-cell activation by halting cytokine production. It inhibits calcineurin. Calcineurin is a calcium and calmodulin-dependent serine/threonine protein phosphatase. It plays a vital role in the activation of T cells [15,19]. Unfortunately, there is not much data on its use and effectiveness in patients with CD. The data mainly suggest its use its indication and maintenance of remission in refractory, steroid-dependent CD. Cyclosporine has several adverse effects, including but not limited to nephrotoxicity, hypertension, hypomagnesemia, tremors, gingivitis, and hirsutism (Table 3) [15,19].

Antibody Treatment

Tumor necrosis factor (TNF) is a group of receptors and proteins that play a pivotal role in the functioning of the human immune system. TNF is secreted in a 17 kD form called transmembrane. It is a nonglycated protein. It is further split by TNF-α-converting enzyme (TACE) metalloproteinase disintegrin proteins to its active form. TACE cleaves the precursor form to transmembrane and soluble TNF-α. TNF-α navigates its action through type 1 and type 2 TNF receptors (TNF-R1 also known as TNFRSF1A, CD120a, and TNF-R2 also known as TNFRSF1B, CD120b, respectively) [16,20]. Transmembrane TNF-α also utilizes TNF-R1 and TNF-R2, but its biological outcome mainly comes from binding to R2. Monocytes, macrophages, and T lymphocytes produce TNF-α at a higher degree, but it is also produced by mast cells, granulocytes, fibroblasts, and several other cell types. TNF is a highly inflammatory cytokine. Its effect manifests as fever, bone reabsorption, insulin resistance, and T-cell activation in response to numerous stimuli, such as bacteria, viruses, immune complexes, stress, malignancy, and radiation [16,20]. The main pathogenesis of CD is the damage of the mucosal barrier, triggering a casket of inflammation, which ultimately does more harm than good by flooding the gut with proinflammatory cytokines, interferon-gamma, and interleukin 12 [16,20]. Therefore, TNF therapy provides relief in such patients by multiple mechanisms. Nullification of TNF-α, reverse signaling, apoptosis, and cytotoxicity are the major mechanisms employed by it. It has a preference for injured tissues and shows good penetration into inflamed tissues. They bind TNF-α, blocking further upregulation of pro-inflammatory molecules and blocking the production of cytokines [16,21-22]. TNF-α is elevated in CD causing excessive immune response and inflammation. This therapy's usefulness has been proven time and again in difficult cases of CD, which cannot be contained by mainstream drugs, such as corticosteroids, methotrexate, and AZA/6-MP. Combining these drugs with immunosuppressive agents has been proven to show better results than using either drug class as monotherapy. Antibody agents can be used both for the suppression of active flare and maintenance therapy [16,21-22].

There are often major questions posed about whether or not to stop these agents once the remission has been achieved. Several studies on this have shown conflicting results, and it is generally advised not to routinely stop these therapy if it is being tolerated well [21,22]. However, if the patient shows any sign of severe infections, several studies have reported reactivation of TB and hepatitis B and C. Patients must be screened for this infection before starting the therapy. Once the infections have been well contained, this therapy can be restarted with careful monitoring of the patients [22]. Patients who come from a high incidence of certain fungal infections should also be screened for systemic fungal infections before the start of antibody treatment. This drug class is strictly contraindicated for patients with a history of hypersensitivity reactions and in patients with NYHA class III or IV congestive heart failure [21,22].

Infliximab

Infliximab is a genetically engineered chimeric (mouse/human) immunoglobulin (Ig)G1 anti-human tumor necrosis factor agent that is administered intravenously. Its main indication is treating active CD unresponsive to another pharmacotherapy [21,22]. Infliximab is also used in the management of active fistulas in CD. Adverse effects include shortness of breath, chest pain, rashes, and severe hyper or hypotension (Table 4). These adverse effects can be present with or without fever and/or chills [21].

**Table 4:** Adverse effects of antibody treatments in CD

Adverse effects of antibody agents	Bandwidth of effects
Malignancies	Increased risk of malignancies, especially lymphomas; black-box warning by the FDA. Non-melanoma skin cancers [20].
Congestive heart failure	Poor outcome in patients with congestive heart failure NYHA class III or IV [20].
Drug-induced lupus	Anti-nuclear antibody (ANA) and anti-Ds-DNA antibodies, skin rash, thrombocytopenia, leukopenia, hypocomplementemia, and hemolytic anemia (rarely) [20].
Demyelinating disorders	Chronic inflammatory demyelinating polyneuropathy, multiple sclerosis (including exacerbation of underlying multiple sclerosis), optic neuritis myelitis, encephalitis, Guillain-Barre syndrome, and transverse myelitis [20].
Skin reactions	Rashes-local injection site erythema, allergic mediated diffuse rash, lupus-like rashes, hypersensitivity vasculitis, urticaria, new-onset psoriasis, granuloma annulare, erythema multiforme, and cutaneous T-cell lymphoma [20].
Pregnancy and lactation	Class B pregnancy risk by the FDA, lack of data on pregnant and lactating females. However, its use in this group should only be considered if the benefit outweighs the risk [20].

Adalimumab

Adalimumab, a monoclonal antibody sourced entirely from humans, uses a complement to destroy cells with TNF-α. It is administered subcutaneously. The goal is to maintain remission in those non-responsive or unable to tolerate Infliximab [21,22]. Adverse effects include tuberculosis (TB), bacterial sepsis, invasive fungal infections, and other opportunistic infections (Table 4). Before planning to start a CD patient on adalimumab therapy, it is strictly advisable to check the patient for TB. Furthermore, rigorous monitoring must be done in any patient with latent TB to prevent its activation or reactivation [21,22].

Certolizumab Pegol

Certolizumab pegol is an anti-TNF with one of its kind structures. Certolizumab pegol lacks an Fc portion. Due to the absence of the Fc portion, there are no Fc-mediated effects [21]. This property of certolizumab pegol makes it different from other monoclonal antibodies that are used in CD management. Due to its conjugation with polyethene glycol, it can increase the half-life of certolizumab. It is only used with patients who were initially compliant with infliximab or adalimumab but wane tolerance toward them or become non-responsive. The unwanted effects stem from the increased risk of opportunistic infections (Table 4) [21].

Anti-Integrin Agents

Anti-integrin agents are similar to their working TNF-α inhibitors in a way that it is specifically designed to target the molecules that result in triggering the inflammatory casket in the intestinal mucosa. However, unlike TNF-α inhibitors, its target is integrin responsible for leukocyte trafficking [15,16]. Integrins are receptor proteins present in cells that aid in cell-to-cell and cell-to-extracellular matrix adhesion. This results in the blockage of leukocyte to migrate to the GI mucosa from vessels and halts the inflammatory domino. The mechanism of action of this drug is directly on α4β7 integrin, without disturbing α4β1 integrin [15,16]. By escaping the binding to the α4β1 integrin, vedolizumab can avoid leukocyte extravasation outside the GI mucosa. The surface of the endothelial present in venule and lymphoid tissue spread in the GI is rich in mucosal vascular addressin cell adhesion molecules. Natalizumab and vedolizumab are the two main anti-integrin agents for patients with CD. Vedolizumab belongs to the family of humanized monoclonal IgG1 antibodies [15,16]. Natalizumab is also a monoclonal antibody that targets the α4-integrin chain, blocking the effect and thereby preventing the interaction between inflammatory cells with VCAM-1 molecules, which are present in vascular endothelial cells. The result is blockage of both the α4β1 and α4β7 integrins [15,16]. Anti-integrin's major advantage over other therapies is its use in blocking the alternative inflammatory pathways in parallel to the use of TNF-α integrin, inhibitors. It can be particularly helpful in groups of patients who show refraction and intolerance toward traditional therapies. The adverse effects profile of this class is dramatically reduced in comparison to drugs that act systemically, particularly if the integrin is only targeted against those expressed in the GI mucosa; such novel anti-integrin therapies are under development [15,16]. Therefore, natalizumab and vedolizumab are used for maintaining remission in those who have achieved remission induced by corticosteroids. However, certain adverse effects have been noted with the use of natalizumab, which includes skin rash, gastrointestinal symptoms, and progressive multifocal leukoencephalopathy (PML) [22,23].

Antibiotics

In order to understand the role of antibiotics when treating patients with CD, it is important to take into consideration that many studies have shown that dysbiosis plays a major role in the development of CD. It is noteworthy that parts of the intestine with the highest microbiome are also the same parts most affected by CD lesions [15,24]. Studies have also repeatedly pointed out that patients suffering from CD have alteration in gut microbiome with an unproportional increase in the population of invasive bacteria such as *Escherichia coli* (*E. coli*). In addition, patients with CD have been observed for their loss of tolerance to the commensal flora [15,24]. The mechanism of action of antibiotics in patients with CD is to eliminate aggressive bacteria, reduce their adherence to the gut mucosa, and minimize bacteria invasion and penetration into the mucosa of the intestine. These desired outcomes can be enhanced by pairing antibiotics with probiotics and by prescribing antibiotics with some degree of immunosuppressive effects [15,24]. However, the use of antibiotics in the management of active CD is highly debated. The usefulness of antibiotics in the treatment of CD is in the treatment of fistulas and abscesses that can present nasty complications of CD. It can be used as a second-line therapy if amino salicylates fail to provide relief. The most commonly used antibiotics are metronidazole, ciprofloxacin, vancomycin, and rifaximin [24-27]. However, the use of antibiotics can also present with some unwanted effects, including *Clostridium difficile* infection, tendonitis, tendon tears, light sensitivity, lack of cartilage growth in unborn babies and children, oral thrush, QT prolongation (a heart rhythm disorder), and several GI issues (Table 5) [15,19].

**Table 5:** Adverse effects of antibiotics use in Crohn's disease

Antibiotics	Adverse effects
Rifaximin	Elevated CRP, rare- joint pain and muscle tightness [24].
Ciprofloxacin	Tendonitis, tendon tears, light sensitivity, lack of cartilage growth in unborn babies and children, oral thrush, QT prolongation (a heart rhythm disorder) [15,24].
Metronidazole	GI issues, permanent nerve damage, nausea, appetite loss, and a metallic taste [15,19].
Vancomycin	*Clostridium difficile* (*C. difficle*) infection, rebound effect in the gut’s mucosa, antibiotic resistance [24].

Novel targeted treatment for CD

**Figure 4:**
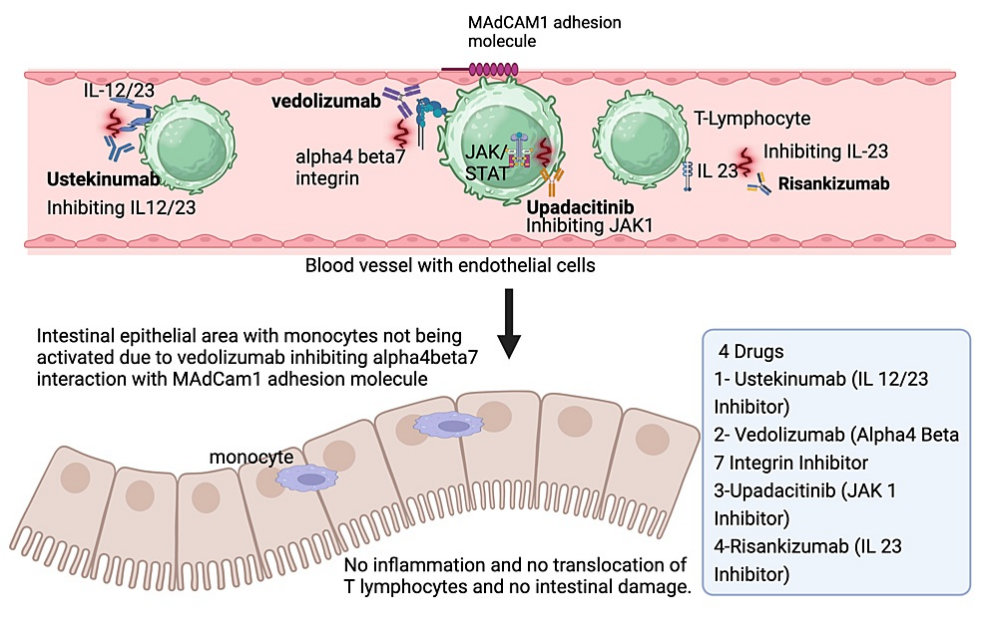
Mechanism of action of various targeted therapies in the management of Crohn’s disease. References:  [16,18,27-30]. Image Credit: Dr. Anam Zara. Created with biorender.com.

In the past few decades, meaningful strides have been made in elucidating treatment modalities targeting specific epigenetic and molecular targets in the management of CD. A few of the noteworthy drugs are discussed in this review, as shown in Figure 4. The molecular targets, mechanism of action, clinical use, and adverse effect profiles of the molecular drug therapies for CD are briefly discussed in Table 6 and elaborated in the respective drug sections.

**Table 6:** Comprehensive overview of molecular drug therapy in Crohn's disease

Drug	Molecular target	Mechanism of action in Crohn’s disease	Clinical use (severity of Crohn's disease used to treat)	Adverse effect
Certolizumab	Tumor necrosis factor-alpha (TNF-alpha)	Monoclonal antibody conjugated to polyethylene glycol that binds TNF-alpha, preventing TNF-alpha mediated inflammatory cascade.	Moderate to severe	Increased risk of opportunistic infection, bacterial sepsis, fever, chills, rashes, muscle aches, and drug-induced lupus (rarely) [15,16,23].
Natalizumab	Alpha-4 integrin	Humanized monoclonal antibody against the alpha-4 subunit of integrin molecules, thereby blocking adhesion and migration of leukocytes in the gut.	Moderate to severe	Progressive multifocal leukoencephalopathy (PML), skin rash, gastrointestinal symptoms including nausea and gastroenteritis [18,19,21].
Risankizumab	Interleukin-23	Target inhibition of IL-23	Moderate to severe	Hypersensitivity reactions, upper respiratory tract infection, inflammation, nasopharyngitis [27].
Upadacitinib	JAK-1	JAK-1 inhibition	Moderate to severe	Lower immunity, blood clots, allergic reactions, malignancy [28].
Ustekinumab	Targets the p40 subunit shared by IL-12 and IL-23	A fully human monoclonal antibody	Moderate to severe	Headache, nasopharyngitis, and upper respiratory tract infections [29].
Vedolizumab	α4β7 integrin	Humanized monoclonal antibody that binds to the α4β7 integrin and blocks the interaction of α4β7 integrin with mucosal addressin cell adhesion molecule-1 (MAdCAM-1)	Moderate to severe	Immunologic: antibody development, headaches, arthralgias, nasopharyngitis, pruritis, and gastrointestinal including nausea [30].

Risankizumab

Interleukin-23 (IL-23) has emerged as a key cytokine implicated in the pathogenesis of CD. IL-23 promotes the differentiation and activation of T helper 17 (Th17) cells, which produce pro-inflammatory cytokines, including interleukin-17 (IL-17), and interleukin-22 (IL-22), driving intestinal inflammation and mucosal damage [31]. Epigenetic modifications can influence the expression of genes involved in IL-23 signaling pathways, modulating immune responses, and contributing to the pathogenesis of CD. In addition, in the short-term study by Boehringer Ingelheim, risankizumab was found to be more effective than a placebo for inducing clinical remission in patients with active CD [31].

Risankizumab is a humanized monoclonal antibody that represents the first and only targeted selective inhibitor of interleukin (IL)-23, targeting the p19 subunit, thereby modulating the inflammatory cascade implicated in CD. Rizankizumab disrupts IL-23-driven immune response, thereby alleviating intestinal inflammation, reducing disease activity, and improving clinical outcomes in patients with CD [32]. It is through different epigenetic changes that genes encoding IL-23 or its receptor components can be expressed differently resulting in the modulation of IL-23-mediated immune responses in CD. Dysregulated transcriptional activity, aberrant DNA methylation patterns, and histone modifications at their promoter regions may change IL-23-related gene’s signaling in CD and hence chronic inflammation [33]. Moreover, further studies are being conducted to see if added inhibition of IL-12 will improve clinical outcomes in CD [32].

The FORTIFY maintenance study by Loftus Jr. et al. and tolerability study by Pang et al. were done to determine the effects of using a body injector for administering risankizumab on patients. Their results showed that patients who were injecting themselves with this drug on the abdomen and upper thighs found it convenient to use and adhere to and mostly did not show any signs of intolerance except in a couple of cases that showed hypersensitivity reactions on the site of injection [34]. To further evaluate OBI, rizankizumab was subcutaneously administered using this device in various volumes and different rates. According to the results, rates and volume have no effect on the amount of this drug that enters the circulation [35]. The study investigated the efficacy and safety of risankizumab in treating CD among Asian patients, utilizing data from three global trials: ADVANCE, MOTIVATE, and FORTIFY, which enrolled a total of 198 participants from various Asian nations. The findings revealed that risankizumab treatment induced clinical remission and endoscopic response by week 12 and sustained effectiveness for up to 52 weeks, aligning with results from non-Asian and global cohorts. Moreover, risankizumab demonstrated positive outcomes in managing fistulizing disease, although the sample size for this subgroup was small. The safety profile of risankizumab mirrored that of non-Asian and global populations, characterized by low rates of serious adverse events and treatment discontinuations. Importantly, no instances of active tuberculosis were reported, suggesting a potential advantage of IL-23 inhibitors over other therapies in regions with endemic tuberculosis. Nonetheless, the analysis faced limitations due to the relatively small number of Asian participants and the absence of a true placebo group during maintenance therapy, potentially affecting the observed efficacy. In conclusion, risankizumab exhibited promising efficacy and safety in CD treatment among Asian patients, presenting it as a viable therapeutic option for this demographic [36].

There was another study by Gao et al., which compared rizankizumab to placebo, and according to the results, rizankizumab given in 600 mg doses subcutaneously was most effective in the management of moderate to severe CD and was considered to be safe and equally effective in all population including Asians. However, fistula closure was seen in 28.6% cases. After successful induction of the first dose, patients were randomly chosen to enter phase 3 of the trial where they were given rizankizumab every eight weeks for the next 52 weeks. In these patients, 180 mg proved to be the most successful dose [36]. Giving fecal calprotectin or CRP in combination with rizankizumab showed better maintenance in 56.6-83.3%. Patients given with rizankizumab through the skin showed better simple endoscopic scores and improved biomarkers [37]. The most frequently reported adverse events were hypersensitivity reactions, upper respiratory tract infections, and nasopharyngitis. In a few cases, it was found that the use of rizankizumab has brought some undesirable adverse effects like inflammation and nasopharyngitis [27].

Targeting the therapeutic of IL-23 using rizankizumab could bring hope to the treatment of CD. By way of selective inhibition of immune responses mediated by IL-23, rizankizumab achieves immune homeostasis reinstatement and relief from intestinally inflamed areas within patients who have CD Furthermore, epigenetic regulation of myelomonocytic leukemia (MML) cluster expression could offer new directions for treatment through modulation of specific genes in this pathway [32].

Upadacitinib

The Janus kinase-signal transducer and activator of transcription (JAK-STAT) signaling pathway plays a pivotal role in mediating inflammatory responses in CD. Dysregulated JAK-STAT signaling pathway activation results in the abnormal production of pro-inflammatory cytokines, such as IL-6, IL-12, and interferon gamma leading to intestinal inflammation and mucosal injury in CD [38]. Upadacitinib is an inhibitor of the JAK-1 enzyme, selectively blocking it, and thereby interfering with JAK-STAT signaling pathways that are linked to the immune responses, which play a role in the pathogenesis of CD. By preventing the synthesis of inflammatory cytokines, inflammation diminishes while the gastrointestinal immune system recovers homeostasis, eventually resulting in an improvement of the condition and better clinical results for CD patients [39].

Inhibition of Janus kinase by upadacitinib has been proven to be successful in the management of moderate to severe CD in several cohort studies and in inducing remission to a level 4 in the Harvey-Bradshaw index [40,41]. These studies explain the clinical effects of this drug by monitoring the frequency of stools passed and by pain in the abdomen described by patients included in the study [41]. A study by Aguilar et al. (2021) investigated the effects of upadacitinib treatment on gene expression in the colon and ileum of patients with CD, focusing on endoscopic response and remission at weeks 12/16. Significant gene regulation was observed in the colon compared to baseline at week 0, with endoscopic remission associated with notable changes in gene expression in the ileum. This difference was more pronounced in the colon. A substantial number of protein-coding genes were significantly regulated in colonic biopsies during endoscopic remission, with 60 genes similarly regulated in both the colon and ileum of patients achieving remission [40]. Trials against CD evaluating upadacitinib’s safety and efficacy have also shown positive outcomes. In phase II and III trials, upadacitinib has been found to be highly effective in achieving and maintaining clinical remission and decreasing markers of inflammation, leading to improved patient-reported outcomes [42]. Several studies have included the observational study by Miyatani et al. labeling upadactnib's safety for use in the treatment of CD after 90% successful results and only a small percentage of patients who experienced discomfort, vomiting, breathing difficulty, and intestinal obstruction [43]. According to the phase 3 induction trial by Loftus Jr., upadacitinib, if taken in doses of 45 or 30 mg, has adverse effects, like herpes zoster, GI perforations, neutropenia, and hepatic disorders, but these risks outweigh the benefits because when compared to placebo, it has the disease under control [44]. From immunodeficiency and opportunistic infections to blood clots and malignancies, there is a wide range of adverse effects from using this medicine [28]. 

Other ongoing studies compare the effectuality between upadacitinib and other biologic agents referred to earlier on like antitumor necrosis factor (TNF) therapies alongside additional JAK inhibitors. These studies aim at better understanding where precisely it should be allocated in the treatment algorithm for CD and thereby guide therapy choices based on the personal characteristics of a patient or targets set out by a doctor [41]. There is a need for extra research to elucidate the long-term safety and efficacy of upadacitinib in real-world clinical practice. In addition, ongoing studies exploring the mechanisms of action of upadacitinib and its potential role in combination therapy regimens may provide insights into novel treatment strategies for patients with refractory CD [41]. 

Ustekinumab IL-12/23 Inhibitors

TNF inhibitors have been around since the late 1990s and were the first drugs approved for treating inflammatory bowel disease. They are valuable because of their role in causing remission to start and remain stable besides reducing complications of illnesses and promoting healing of the mucosal surface. However, as many as 30% of patients do not get better after the first round of therapy, 50% become worse over time, and 10% need surgery [45]. Consequently, novel pharmacological approaches focusing on distinct routes of inflammation in the intestines were required. Efforts are underway to create novel drugs targeting pathways, including α4β7 integrin, JAK, and IL-12/IL-23. As an example, the fully human monoclonal antibody ustekinumab (UST) blocks the interaction between the common receptor IL-12Rβ1 and the IL-12/IL-23 p40 subunit. This approach lessens the inflammatory processes that cause CD pathogenesis and decreases cytokine production [45,46].

In addition, UST, an antibody that targets the p40 subunit shared by IL-12 and IL-23, was more efficient than a placebo in improving endoscopic and clinical results in patients with moderate to severe CD. Thus, this biological therapy may also protect against postoperative recurrence [46]. The FDA has approved UST to treat inflammatory bowel diseases. The heterodimeric cytokine IL-12 consists of two subunits, p35 and p40. Dendritic cells, macrophages, and neutrophils produce it when exposed to antigens; nonetheless, it was first identified as a mitogen-induced factor that stimulates the growth of natural killer (NK) cells. Colitis is one of many immune-mediated diseases that have responded well to blocking IL-12 p40 in animal studies. A new interleukin, IL-23, was found to include the same p40 and a different p19 subunit; however, focusing on only the p35 subunit did not provide the same results and, in many instances, worsened inflammation. Important mediators of CD include IL-23 rather than IL-12. It may trigger inflammation when IL-23 interacts with macrophages, innate lymphoid cells, and pathogenic TH17 cells. However, IL-23's protective effects may be enhanced by epithelial cells, antimicrobial pathways, and TH1 cell downregulation. Therefore, CD treatment may need the targeted inhibition of this cytokine [47-49].

The pharmacokinetics of UST have been looked into a great deal. Since the drug looks like human IgG1, it can be injected intravenously (IV) or subcutaneously (SC). Both methods show that the half-life of elimination is about three weeks long. The newborn Fc receptor keeps antibody proteins from being broken down by lysosomes. This is what makes the clearance last longer [50]. There are some differences in how UST works in different people though. Several things can affect how the drug works in the body, including the level of inflammation, the type of disease, the blood albumin concentration, the person's body mass index, and any immunosuppressants they are taking at the same time [50].

On the other hand, the UST dosing plan looks at how well the patient reacts to the first treatment to figure out the maintenance dose. Beginning in week 0, the first dose is usually given directly at a rate of about 6 mg/kg. In week 8, 90 mg amount is given under the skin. After that, 90 mg is injected under the skin as needed for upkeep every eight to 12 weeks, based on how the patient reacts. At 16 weeks, patients are checked to see if they are responding well enough. Those who should switch to a 12-weekly dose, while those who are only partly responding should keep up the eight-weekly treatment. It might be okay to keep up the eight-weekly dose past week 16 in case the response is slow, but people whose symptoms are not improving or worsening should still think about stopping the drug. It might be helpful to compare the Harvey-Bradshaw Index (HBI) scores at the starting point and before week 16 with objective markers of inflammation (CRP and fecal calprotectin) [51].

Several meta-analyses show that many patients who get UST treatment may not respond well or at all after initially responding well. This is especially true for those who have been exposed to TNFα inhibitors before. Because of this, UST dosage increase, which includes re-induction and shorter time between doses, is often used [52]. A review of 15 cohort studies found that more than half of the 925 CD patients who were given UST and had their doses raised got better. About 40% of them reached clinical remission without using corticosteroids. Dose escalation resulted in endoscopic response in around 61% of patients and endoscopic remission in about 29%. The majority of these trials' escalation strategies included decreasing the time between doses to every four to six weeks. Patients with refractory CD, especially those showing a partial response to treatment, might greatly benefit from the UST dosage increase, as shown in these results [52].

The effectiveness of the CD medicine UST depends on several individual circumstances. However, whether UST treatment is suitable for a patient depends on several characteristics that are specific to their condition. When it comes to CD, the patient's age is one of the most important variables that might affect the prognosis and the results of therapy. The clinical response to UST may be less in older individuals, particularly in the near term. Gender and ethnicity are two more sociodemographic factors that should be considered while thinking about patients. Research shows that UST is more effective in treating white patients and female patients than other racial groupings [52]. Another crucial factor is the patient's weight, as studies have revealed that the treatment with anti-TNF works better in patients with lower body weights. The loading dosage of UST that is presently allowed is dependent on weight, and there has been evidence that individuals with a lower body weight tend to have better clinical response rates throughout the induction phase. Before starting UST treatment, it is important to determine the severity of the condition according to the Montreal classification. When the colon is the only site of infection, good results have been seen. However, the response to biologics is greatly affected by problems like structural or penetrating issues. The gut microbiota has a vital role in the development of CD, and it has been shown to have predictive power when it comes to the efficacy of some medical treatments and may even have a correlation with treatment response [52].

In addition, studies have shown that the levels of biological medications in the bloodstream are inversely correlated with inflammation levels [53]. Patients in remission have significantly higher serum drug levels than those with ongoing inflammation. One reason for this relationship is the loss of protein that often occurs during severe intestinal inflammation. Since biological therapies consist of molecules of similar sizes, this phenomenon is likely to apply broadly across this class of medication [53]. The American Gastroenterological Association's Technical Review on the Medical Management of Moderate to Severe Crohn's Disease, either limited to lumen or with fistulas, assessed five studies on combination biological therapy for juvenile inflammatory bowel disease. The study found that biological therapy helps youngsters with active inflammatory bowel disease. After six months, 75% of pediatric patients in the most extensive study group achieved steroid-free clinical remission. On the other hand, two patients had to switch up their treatment plans, and one patient needed surgery [54].

Moreover, the impact of UST usage before surgery on postoperative results in CD patients has only been examined in three published studies. One research had participants from the United States, Italy, and Belgium, and it was a multicentric experience. A case-control study conducted in Canada was the second research. Mayo Clinic researchers most recently looked at how UST affected postoperative outcomes in a retrospective single-center cohort study. According to the authors of all three trials, patients with CD may safely use UST throughout the perioperative phase [29]. A positive safety profile for UST was found in clinical studies with more than 3,000 participants. Research on psoriasis, MS, and CD provides most of the data. There was no statistically significant difference between the placebo and UST in terms of the occurrence of major adverse events, as reported by Toussirot et al. (2013). Headache, nasopharyngitis, and upper respiratory tract infections are among the everyday adverse effects recorded during five years of treatment. Malignancy occurrence was not more significant than that of the general population when nonmelanoma skin cancers were excluded. There were no recorded instances of TB. However, the most common significant adverse event was a severe infection. Nevertheless, clinical testing did reveal a few cases of heart problems. In clinical trials including psoriasis patients, there was a numerical imbalance in significant cardiovascular events between the UST and placebo groups. However, in phase III UNITI investigations, no cardiac events were recorded [26].

Lastly, UST should be used with extreme caution by pregnant and nursing mothers. Human data is scarce, despite the lack of evidence linking UST exposure to congenital abnormalities in cynomolgus monkey research [55]. Pregnant women should exercise care and not use UST. Furthermore, whether or not the medicine is excreted in human breast milk is uncertain. Women should consider the possible adverse effects of UST on nursing babies before deciding to breastfeed. They need to consider these variables when deciding whether to cease breastfeeding while on the treatment and up to 15 weeks following treatment or to end therapy altogether [55].

Vedolizumab (Integrin Inhibitors)

Integrins are signaling and cell adhesion proteins that are essential to cellular function. They allow binding of cell-surface ligands, soluble ligands, and extracellular matrix ligands. Integrins are transmembrane glycoproteins that consist of an α-subunit and a β-subunit that are non-covalently bound [56]. Up to date, there are at least 18α and 8β subunits in humans, of which 8α and 8β subunits can signal across the plasma membrane of cells bidirectionally. Integrins are the principal receptors found on cells and can bind most extracellular matrix proteins, such as collagen, laminins, and fibronectin. This allows integrins to respond to extracellular environmental changes. Intracellular communication upon ligand binding results in cytoskeletal changes, changes in gene expression, and cellular proliferation [56]. Virtually, every cell within multicellular organisms expresses these heterodimeric transmembrane receptors, which mediate this bidirectional signaling between the extracellular environment and the intracellular environment. Throughout the extracellular-intracellular signaling, the binding of ligands to integrins initiates intracellular signaling pathways. These lead to downstream processes, such as cellular proliferation, apoptosis, gene expression, cell migration, protein phosphorylation, and differentiation [56].

The general DNA sequence motif of integrins is arginine-glycine-aspartic acid (RGD), but there are individual integrins specifically for certain protein ligands. Within the immune system, integrin ligands are the intercellular adhesion molecules (ICAMs) that are present on antigen-presenting cells (APCs) and inflamed endothelium. Upon binding with the ligand, integrins work to transduce signals intracellularly. In addition, integrins can receive intracellular signals that allow for regulating their ligand-binding affinity [57]. Integrins are typically classified into four types: leukocyte cell-adhesion integrins, arginine-glycine-aspartic acid (RGD)-binding integrins, collagen-binding integrins, and laminin-binding integrins [58]. The function of integrins depends on a balance between both the inactive and active mechanisms, such as conformational changes, trafficking, and protein-protein interactions. These mechanisms are activated via “inside-out” and “outside-in” signals. Activation occurs via the binding of integrins with the extracellular matrix ligands. Downstream signaling mediators include Src-family protein tyrosine kinases, integrin-linked kinase (ILK), and focal adhesion kinase (FAK) [59]. Activation by integrins can be done intracellularly via G-protein-coupled receptors (GPCRs) that lead to downstream phosphorylation β subunit cytoplasmic domain. Chemokines allow for integrin activation, which then signals via GPCR [60]. When integrins are bound to an extracellular ligand, they generate intracellular signals that allows cell function to be regulated by signals within the cell itself. They are the transmembrane linkage between the actin microfilaments of the cell cytoskeleton and the extracellular matrix. Binding triggers events of signal transduction events that modulate cellular gene expression, polarity, proliferation, and cellular adhesion. Integrins also play a critical role in vasculogenesis via αV integrins, lymphangiogenesis via α9β1, development via β1, immune response via β2, and thrombus formation via αIIβ3 integrins [61].

Integrins have also shown a role in wound healing and in suppressing inflammation and atherosclerosis [62]. The adaptive and innate immune responses depend on immune cell trafficking to a specific disease-target organ. Inflammatory activation results in circulating leukocyte migration to these target-specific organs. The process of migration occurs via interaction between adhesion molecules expressed on both leukocytes and endothelial cells [63]. Leukocytes travel within the bloodstream and roll at a specific site of the vessel wall where they undergo activation, arrest, and adhesion, to the vascular endothelial cells. Cellular adhesion molecules (CAMs), expressed on leukocytes, known as integrins, allow this interaction [63]. Advancements in research in the pathogenesis of CD have led to a shift in treatment from conventional therapies, such as glucocorticoids, immunomodulators, and aminosalicylates toward biological drugs that specifically target inflammatory pathways [64]. The therapeutic initiative in the treatment of CD has progressively changed from symptom control and clinical remission and is now aimed at mucosal remission, histological healing, and transmural healing. Approved therapies also come with considerable limitations, many of which demonstrate only moderate efficacy and are frequently associated with an increased risk of adverse events [65].

Persistent and chronic inflammation in CD, mediated primarily through the migration of pro-inflammatory T-cells into the gastrointestinal tract, is the hallmark of disease progression. T-cell infiltration within the gut depends specifically on the interaction between mucosal addressing cell adhesion molecule (MAdCAM-1) present on endothelial cells and surface-expressed α4β7 integrins [66]. This interaction results in the extravasation of T-cells into the gastrointestinal tract, and hence, drugs targeting α4β7 integrins have become a focal point for targeted therapy. Anti-integrin pharmacotherapy for the treatment of CD inhibits the extravasation of leukocytes by blocking the interaction between integrins and endothelial cell adhesion molecules found on immune cells. Inhibition of the interaction between the intestinal vasculature and leukocytes is achieved via anti-integrin agents, furthermore selectively preventing the influx of inflammatory cells that are responsible for the inflammatory process in CD. Antibodies that block integrin function prevent firm adhesion of leukocytes to the endothelium [66].

Natalizumab is an anti-α4 integrin antibody that prevents leukocyte trafficking systemically and prevents binding to both mucosal adressin cell adhesion molecule-1 and to vascular cell adhesion molecule-1 [67]. It has been proven to be effective in patients who have failed pharmacotherapeutic management with other agents, but natalizumab carries the risk of progressive multifocal leukoencephalopathy (PML), which is caused by the JC virus. Due to the risk of PML, therapy is reserved for the maintenance of natalizumab-induced remission of CD only if the serum antibody to the JC virus is negative. Anti-JC virus antibody is recommended to be repeated once every six months and treatment should be immediately discontinued with positive results [67].

Vedolizumab (VDZ) is a gut-selective humanized immunoglobulin IgG1 monoclonal antibody that has become an integral choice in the management of patients with moderate to severe ulcerative colitis and CD. Vedolizumab is specifically an α4β7 integrin antagonist and inhibits the intestinal translocation of T-cells by blocking the integrin interactions with mucosal vascular address cellular adhesion molecule 1 (MAdCAM1), a cell adhesion molecule selectively expressed in the digestive tract circulatory system. Therefore, this blockage results in a reduction of lymphocyte-mediated inflammation and inhibition of lymphocytes in the gut [63]. The gastrointestinal-specific tropism of the α4β7 integrin subunit creates a beneficial drug profile and therefore does not involve systemic immunosuppression [56]. VDZ has shown to be effective for patients who are actively symptomatic with CD regardless of prior treatment with anti-TNF agents, methotrexate, thiopurines, and corticosteroids. Therapy with VDZ has been shown to achieve clinical remission and corticosteroid-free remission in patients [67]. However, mandatory failure with other agents for the treatment of CD is not required before initiation of VDZ. Unlike natalizumab, one of the promising features of VDZ is that the drug is not associated with progressive multifocal leukoencephalopathy(PML) [68].

Research has shown that response to VDZ can be predicated using individual biomarkers. Before treatment, expression of α4β7 integrin on multiple immune cells showed a higher rate of response to VDZ. In addition, serum retinoic acid levels obtained before initiating treatment with VDZ were predictive of achieving clinical remission [58]. Moreover, achieving clinical remission with VDZ was associated with nearly undetectable levels of soluble MAdCAM-1 in maintenance therapy [69]. Furthermore, potential biomarkers such as α4β7 integrin, and soluble-MAd-CAM-1 may be useful in predicting remission during treatment [70]. This may lead to more personalized therapy for patients with inflammatory bowel disease. Integrin inhibitors, such as VDZ, are essential for moderate to severe ulcerative colitis and CD. The gut specificity of VDZ and its efficacy make the drug a first-line agent for both induction and maintenance of remission in patients with inflammatory bowel disease. Although the response to VDZ for the treatment of CD is promising, the lack of response is still seen in a subset of patients, which may be due to the lack of efficacy and may be multifactorial, which may render the mechanism of action of the drug ineffective. Newer biologics can effectively decrease surgical rates and hospitalization in patients with CD due to their target-specific actions and anti-inflammatory mechanisms. They have been demonstrated to be advantageous in terms of good efficacy with a lower incidence of adverse effects and therefore a higher level of safety in patients [68]. Adverse effects range from less severe headaches, arthralgia, nasopharyngitis, injection-site reactions, fatigue, and gastrointestinal symptoms to immunologic reactions including antibody development [30].

## Conclusions

Newer CD treatment options like risankizumab, upadacitinib, ustekinumab, and vedolizumab, targeting specific steps in CD management, have opened a landscape of clinical remission and improved patient outcomes. The focus on precision medicine, epigenetic regulation, and individualized biomarkers opens doors for personalized therapeutic strategies. With continuing research on safer and more effective treatment options, the importance of molecular pathways for CD management is reinforced. The future efforts in the field of targeted therapies for treating CD would depend on personalized treatment approaches as per individual patient characteristics like genetic markers, disease phenotype immune profile, and microbiome composition. Moreover, there is a promise for combining synergistic mechanisms of action to increase effectiveness, decrease drug resistance, or reduce side effects. Long-term safety studies and rigorous monitoring protocols are essential to assess and mitigate potential risks associated with targeted therapies, but patient-centered outcomes research can shed light on some aspects like quality of life and treatment satisfaction. Comprehensive cost-effectiveness analyses will be important to determine the economic value of these advanced therapies compared to conventional approaches. By pursuing these avenues, the field can advance toward more effective, personalized, and sustainable management of CD.

## References

[1] Nakase H, Uchino M, Shinzaki S (2021). Evidence-based clinical practice guidelines for inflammatory bowel disease 2020. J Gastroenterol.

[2] Gettler K, Levantovsky R, Moscati A (2021). Common and rare variant prediction and penetrance of IBD in a large, multi-ethnic, health system-based biobank cohort. Gastroenterology.

[3] Larabi A, Barnich N, Nguyen HT (2020). New insights into the interplay between autophagy, gut microbiota and inflammatory responses in IBD. Autophagy.

[4] Annese V (2020). Genetics and epigenetics of IBD. Pharmacol Res.

[5] Neurath MF, Vieth M (2023). Different levels of healing in inflammatory bowel diseases: mucosal, histological, transmural, barrier and complete healing. Gut.

[6] Dulai PS, Singh S, Vande Casteele N (2019). Should we divide Crohn's disease into ileum-dominant and isolated colonic diseases?. Clin Gastroenterol Hepatol.

[7] Greuter T, Piller A, Fournier N (2018). Upper gastrointestinal tract involvement in Crohn's disease: frequency, risk factors, and disease course. J Crohns Colitis.

[8] Jarmakiewicz-Czaja S, Zielińska M, Sokal A, Filip R (2022). Genetic and epigenetic etiology of inflammatory bowel disease: an update. Genes (Basel).

[9] Palandurkar GS, Kumar S (2023). Biofilm's impact on inflammatory bowel diseases. Cureus.

[10] Marks DJ, Rahman FZ, Sewell GW, Segal AW (2010). Crohn's disease: an immune deficiency state. Clin Rev Allergy Immunol.

[11] Mikami Y, Tsunoda J, Suzuki S, Mizushima I, Kiyohara H, Kanai T (2023). Significance of 5-aminosalicylic acid intolerance in the clinical management of ulcerative colitis. Digestion.

[12] Cheifetz A, Cullen G (2024). Sulfasalazine and 5-aminosalicylates in the treatment of inflammatory bowel disease. UpToDate.

[13] Bruscoli S, Febo M, Riccardi C, Migliorati G (2021). Glucocorticoid therapy in inflammatory bowel disease: mechanisms and clinical practice. Front Immunol.

[14] Choi J, Patel P, Fenando A (2024). Sulfasalazine. StatPearls [Internet].

[15] Kumar A, Cole A, Segal J, Smith P, Limdi JK (2022). A review of the therapeutic management of Crohn's disease. Therap Adv Gastroenterol.

[16] Gade AK, Douthit NT, Townsley E (2020). Medical management of Crohn's disease. Cureus.

[17] Barrett K, Saxena S, Pollok R (2018). Using corticosteroids appropriately in inflammatory bowel disease: a guide for primary care. Br J Gen Pract.

[18] Hodgens A, Sharman T (2023). Corticosteroids. StatPearls [Internet].

[19] Randall CW, Vizuete JA, Martinez N, Alvarez JJ, Garapati KV, Malakouti M, Taboada CM (2015). From historical perspectives to modern therapy: a review of current and future biological treatments for Crohn's disease. Therap Adv Gastroenterol.

[20] Adegbola SO, Sahnan K, Warusavitarne J, Hart A, Tozer P (2018). Anti-TNF therapy in Crohn's disease. Int J Mol Sci.

[21] Yamazaki H, So R, Matsuoka K (2017). Certolizumab pegol for induction of remission in Crohn's disease. Cochrane Database Syst Rev.

[22] Hazel K, O'Connor A (2020). Emerging treatments for inflammatory bowel disease. Ther Adv Chronic Dis.

[23] McLean LP, Cross RK (2016). Integrin antagonists as potential therapeutic options for the treatment of Crohn's disease. Expert Opin Investig Drugs.

[24] Nitzan O, Elias M, Peretz A, Saliba W (2016). Role of antibiotics for treatment of inflammatory bowel disease. World J Gastroenterol.

[25] Scribano ML, Prantera C (2013). Use of antibiotics in the treatment of Crohn's disease. World J Gastroenterol.

[26] Vaughn BP (2021). A practical guide to therapeutic drug monitoring of biologic medications for inflammatory bowel disease. J Clin Med.

[27] Horst S, Cross RK (2023). Clinical evaluation of risankizumab in the treatment of adults with moderately to severely active Crohn's disease: patient selection and reported outcomes. Drug Des Devel Ther.

[28] Wodeyar AM, Pansuriya N, Saeed S (2023). Upadacitinib in Crohn's disease: a comprehensive systematic review of efficacy and safety. Cureus.

[29] Quaresma AB, Yamamoto T, Kotze PG (2020). Biologics and surgical outcomes in Crohn's disease: is there a direct relationship?. Therap Adv Gastroenterol.

[30] Colombel JF, Sands BE, Rutgeerts P (2017). The safety of vedolizumab for ulcerative colitis and Crohn's disease. Gut.

[31] Feagan BG, Sandborn WJ, D'Haens G (2017). Induction therapy with the selective interleukin-23 inhibitor risankizumab in patients with moderate-to-severe Crohn's disease: a randomised, double-blind, placebo-controlled phase 2 study. Lancet.

[32] Johnson A, Loftus E Jr (2023). Risankizumab to treat moderately to severely active Crohn's disease in adults: an evaluation of trials and data. Expert Rev Gastroenterol Hepatol.

[33] Xu S, Li X, Zhang S (2023). Oxidative stress gene expression, DNA methylation, and gut microbiota interaction trigger Crohn's disease: a multi-omics Mendelian randomization study. BMC Med.

[34] Loftus EV Jr, Griffith J, Neimark E (2023). Efficacy, safety, patient experience, and tolerability of risankizumab administered by on-body injector for moderate to severe Crohn's disease. Adv Ther.

[35] Pang Y, D'Cunha R, Mohammad AS (2024). Clinical bridging from prefilled syringe to on-body injector for risankizumab in Crohn's disease. Clin Ther.

[36] Gao X, Fujii T, Ye BD (2024). Efficacy and safety of risankizumab for Crohn's disease in patients from Asian countries: a post hoc subanalysis of the global phase 3 ADVANCE, MOTIVATE, and FORTIFY studies. J Gastroenterol Hepatol.

[37] Ferrante M, Irving PM, Abreu MT (2024). Maintenance risankizumab sustains induction response in patients with Crohn's disease in a randomized phase 3 trial. J Crohns Colitis.

[38] Neurath MF (2014). Cytokines in inflammatory bowel disease. Nat Rev Immunol.

[39] Sandborn WJ, Feagan BG, Loftus EV Jr (2020). Efficacy and safety of upadacitinib in a randomized trial of patients with Crohn's disease. Gastroenterology.

[40] Aguilar D, Revilla L, Garrido-Trigo A (2021). Randomized controlled trial substudy of cell-specific mechanisms of Janus kinase 1 inhibition with upadacitinib in the Crohn's disease intestinal mucosa: analysis from the CELEST study. Inflamm Bowel Dis.

[41] Chugh R, Braga-Neto MB, Fredrick TW (2023). Multicentre real-world experience of upadacitinib in the treatment of Crohn's disease. J Crohns Colitis.

[42] Törüner M, Ünal NG (2023). Epigenetics of inflammatory bowel diseases. Turk J Gastroenterol.

[43] Miyatani Y, Choi D, Choi NK, Rubin DT (2024). Dual-targeted therapy with upadacitinib and ustekinumab in medically complex Crohn's disease. Dig Dis Sci.

[44] Loftus EV Jr, Panés J, Lacerda AP (2023). Upadacitinib induction and maintenance therapy for Crohn's disease. N Engl J Med.

[45] Chenna VS, Nagi TK, Suarez ZK (2023). Comparison of effectiveness and safety of ustekinumab and adalimumab as induction or maintenance therapy in patients with moderate to severe Crohn's disease: a systematic review and meta-analysis. Cureus.

[46] Gisbert JP, Chaparro M (2023). Anti-TNF agents and new biological agents (vedolizumab and ustekinumab) in the prevention and treatment of postoperative recurrence after surgery in Crohn's disease. Drugs.

[47] Parigi TL, Iacucci M, Ghosh S (2022). Blockade of IL-23: what is in the pipeline?. J Crohns Colitis.

[48] Koh SJ, Hong SN, Park SK (2023). Korean clinical practice guidelines on biologics for moderate to severe Crohn's disease. Intest Res.

[49] Ashraf H, Bodapati A, Hanif A, Okafor DK, Katyal G, Kaur G, Khan S (2023). Safety and efficacy of biologic therapies (ustekinumab and vedolizumab) in the treatment of inflammatory bowel disease (IBD): a systematic review. Cureus.

[50] Rubín de Célix C, Chaparro M, Gisbert JP (2022). Real-world evidence of the effectiveness and safety of ustekinumab for the treatment of Crohn's disease: systematic review and meta-analysis of observational studies. J Clin Med.

[51] Yzet C, Brazier F, Sabbagh C, Fumery M (2022). Managing complex perianal disease after anti-TNF failure: where to go next?. Curr Res Pharmacol Drug Discov.

[52] Alsoud D, Vermeire S, Verstockt B (2022). Biomarker discovery for personalized therapy selection in inflammatory bowel diseases: challenges and promises. Curr Res Pharmacol Drug Discov.

[53] Singh S, Proctor D, Scott FI, Falck-Ytter Y, Feuerstein JD (2021). AGA technical review on the medical management of moderate to severe luminal and perianal fistulizing Crohn's disease. Gastroenterology.

[54] Lauro R, Mannino F, Irrera N (2021). Pharmacogenetics of biological agents used in inflammatory bowel disease: a systematic review. Biomedicines.

[55] Tamilarasan AG, Cunningham G, Irving PM, Samaan MA (2019). Recent advances in monoclonal antibody therapy in IBD: practical issues. Frontline Gastroenterol.

[56] Slack RJ, Macdonald SJ, Roper JA, Jenkins RG, Hatley RJ (2022). Emerging therapeutic opportunities for integrin inhibitors. Nat Rev Drug Discov.

[57] Kakuta Y, Naito T, Kinouchi Y, Masamune A (2023). Current status and future prospects of inflammatory bowel disease genetics. Digestion.

[58] Kamranvar SA, Rani B, Johansson S (2022). Cell cycle regulation by integrin-mediated adhesion. Cells.

[59] Humphries JD, Chastney MR, Askari JA, Humphries MJ (2019). Signal transduction via integrin adhesion complexes. Curr Opin Cell Biol.

[60] Sun H, Lee HS, Kim SH (2023). IL-2 can signal via chemokine receptors to promote regulatory T cells' suppressive function. Cell Rep.

[61] Besendorf L, Müller TM, Geppert CI (2022). Vedolizumab blocks α4β7 integrin-mediated T cell adhesion to MAdCAM-1 in microscopic colitis. Therap Adv Gastroenterol.

[62] Li S, Sampson C, Liu C, Piao HL, Liu HX (2023). Integrin signaling in cancer: bidirectional mechanisms and therapeutic opportunities. Cell Commun Signal.

[63] Dustin ML (2019). Integrins and their role in immune cell adhesion. Cell.

[64] Coskun M, Steenholdt C, de Boer NK, Nielsen OH (2016). Pharmacology and optimization of thiopurines and methotrexate in inflammatory bowel disease. Clin Pharmacokinet.

[65] Vaughan R, Tjandra D, Patwardhan A (2022). Toward transmural healing: Sonographic healing is associated with improved long-term outcomes in patients with Crohn's disease. Aliment Pharmacol Ther.

[66] Fu S, Ni T, Zhang M (2024). Cholinergic anti-inflammatory pathway attenuates acute liver failure through inhibiting MAdCAM1/α4β7-mediated gut-derived proinflammatory lymphocytes accumulation. Cell Mol Gastroenterol Hepatol.

[67] Chandar AK, Singh S, Murad MH, Peyrin-Biroulet L, Loftus EV Jr (2015). Efficacy and safety of natalizumab and vedolizumab for the management of Crohn's disease: a systematic review and meta-analysis. Inflamm Bowel Dis.

[68] Paul S, Williet N, Di Bernado T (2018). Soluble mucosal addressin cell adhesion molecule 1 and retinoic acid are potential tools for therapeutic drug monitoring in patients with inflammatory bowel disease treated with vedolizumab: a proof of concept study. J Crohns Colitis.

[69] Battat R, Dulai PS, Vande Casteele N (2019). Biomarkers are associated with clinical and endoscopic outcomes with vedolizumab treatment in ulcerative colitis. Inflamm Bowel Dis.

[70] Liu J, Di B, Xu LL (2023). Recent advances in the treatment of IBD: targets, mechanisms and related therapies. Cytokine Growth Factor Rev.

